# Effects of Water–Nitrogen Coupling on Root Distribution and Yield of Summer Maize at Different Growth Stages

**DOI:** 10.3390/plants14091278

**Published:** 2025-04-22

**Authors:** Yanbin Li, Qian Wang, Shikai Gao, Xiaomeng Wang, Aofeng He, Pengcheng He

**Affiliations:** School of Water Conservancy, North China University of Water Resources and Electric Power, Zhengzhou 450046, China; liyb101@163.com (Y.L.); 201201804@stu.ncwu.edu.cn (Q.W.); 17513360713@163.com (X.W.); 18790639808@163.com (A.H.); ihepengcheng@163.com (P.H.)

**Keywords:** correlation analysis, numerical simulation, root distribution, root water uptake, water–nitrogen coupling, yield

## Abstract

This research investigates the influence of water–nitrogen coupling on soil water content, nitrogen dynamics, and root distribution in farmland, along with the interactions among soil water, nitrogen transport, root distribution, and crop yield. A field experiment was conducted under moderate drought stress (50–60% of field capacity) and three nitrogen application rates (100, 200, and 300 kg·ha^−1^, split-applied at 50% during sowing and 50% at the jointing stage, labeled as N_1_, N_2_, and N_3_) at the two critical growth stages (jointing stage P_1_ and tasseling-silking stage P_2_) of maize (*Denghai 605*). The results demonstrated that maize root morphological parameters exhibited the trend N_2_ > N_1_ > N_3_ under different nitrogen treatments. Compared to N_2_, low nitrogen (N_1_) decreased root morphological parameters by 35.01–49.60% on average, whereas high nitrogen (N_3_) led to a reduction of 49.93–61.37%. The N_2_ treatment consistently maintained greater water uptake, with the highest yield of 13,336 kg·ha^−1^ observed under the CKN_2_ treatment, representing increases of 16.1% and 9.2% compared to the P_1_N_2_ and P_2_N_2_ treatments, respectively. Drought stress at the jointing stage (P_1_) inhibited root development more severely than at the tasseling-silking stage (P_2_), demonstrating a bidirectional adaptation strategy characterized by deeper vertical penetration under water stress and increased horizontal expansion under nitrogen imbalance. Correlation analysis revealed a positive correlation between soil nutrient content and maize yield indicators. At the same time, root characteristic values were significantly negatively correlated with yield (*p* < 0.05). Appropriate water–nitrogen management effectively stimulated root growth, mitigated nitrogen leaching risks, and improved yield. These findings offer a theoretical foundation for optimizing water and nitrogen management in maize production within the Yellow River Basin.

## 1. Introduction

As one of the three primary staple crops globally, maize is vital in China’s agricultural sector, with the largest cultivation area and highest total yield [1]. It fulfills multiple functions, including use as food, feed, and industrial raw material, contributing to national food security and the industrial economy [2]. Agriculture currently faces dual challenges of soil nitrogen accumulation and declining planting efficiency [3,4]. Proper nitrogen application can substantially improve maize yield and nitrogen use efficiency while mitigating environmental risks [5]. Water–nitrogen interactions exhibit a threshold effect, where excessive irrigation increases water resource consumption and accelerates nitrogen leaching, while high nitrogen fertilization intensifies water stress. Both factors negatively affect maize growth and yield [6,7]. A well-balanced integration of water and nitrogen fosters a positive feedback loop, in which fertilization enhances water utilization, and water availability promotes nutrient uptake. Thus, optimizing water–nitrogen management is essential for achieving environmentally sustainable agricultural development.

Roots serve as the primary organs through which crops absorb water and assimilate nutrients from the soil, playing a critical role in plant growth and grain production [8]. Crops adjust root distribution and growth patterns in response to neighboring plants and soil nutrient availability, typically exhibiting avoidance, excessive proliferation, or a lack of response [9]. Under abundant soil nutrients, rapid root proliferation occurs, whereas nutrient deficiency restricts root growth and stimulates lateral root formation [10,11]. Zhang et al. identified a highly significant correlation between root traits in deeper soil layers and crop water consumption [12]. Ma et al. [13] reported that increasing nitrogen fertilizer application significantly enhances maize root development in surface soil. However, a comparative study by Wang et al. [14] indicated that higher nitrogen application has a more pronounced effect on increasing root biomass allocation in deeper soil layers. Consequently, examining the dynamic responses of the maize root system under water–nitrogen coupling conditions is of great scientific importance for optimizing farmland water and fertilizer management, improving water use efficiency, and maximizing maize yield.

Water is a fundamental physiological and ecological factor influencing plant growth and development. Both drought and flooding stresses directly affect root growth and morphological structure while regulating photosynthetic product accumulation and distribution [15,16]. Plants adapt to environmental fluctuations through internal physiological processes and external morphological adjustments [17]. Drought stress induces multi-level effects on maize, affecting both morphological development and essential metabolic pathways. Water deficit during critical growth stages leads to yield losses ranging from 21% to 50%, with complete plant mortality occurring under severe drought conditions [18,19]. In contrast to drought, flooding stress primarily affects plants through hypoxic soil environments. These water-saturated soils restrict root aerobic respiration, diminishing metabolic capacity [20]. Consequently, root growth declines significantly, absorption ability weakens, and nutrient uptake is impaired. Additionally, water stress elevates the risk of nitrogen loss through denitrification and soil leaching [21], decreasing nutrient use efficiency. This causes substantial resource waste and contributes to pollution of the agricultural ecosystem.

Nitrogen, as an essential nutrient for crop growth, plays a decisive role in determining yield potential [22]. The application of nitrogen fertilizer can significantly enhance crop productivity; however, inefficient fertilization management and inappropriate application methods, such as broadcasting and surface application, remain prevalent in agricultural practices, leading to various challenges [23]. Research indicates that excessive nutrient uptake does not necessarily contribute to higher yields but can instead inhibit plant growth, reducing productivity and resulting in nutrient wastage [24]. In agricultural systems, nitrogen fertilizers undergo nitrification and urease processes, converting into nitrate (NO_3_^−^) and ammonium (NH_4_^+^) nitrogen, which crops absorb. However, a portion of NO_3_^−^ and NH_4_^+^ salts is lost from the soil through leaching, denitrification, ammonia volatilization, and NO_2_^−^ chemical decomposition, with most nitrogen persisting in the soil as nitrate [25,26]. Under such conditions, improper irrigation exacerbates nitrogen leaching, increasing environmental risks and contributing to groundwater contamination.

Given the intrinsic relationship between water and nitrogen, a scientifically optimized water–nitrogen management strategy is essential for regulating plant nutrient absorption and distribution. However, existing research on water–nitrogen coupling predominantly emphasizes the aboveground responses of crops such as rice, cotton, and maize [27,28,29,30]. In contrast, studies on maize root spatial distribution and its role in regulating water and nitrogen movement remain limited. In particular, research on heterogeneous root layer response mechanisms and nutrient loss is insufficient. Therefore, this study examines maize under a furrow irrigation system, focusing on the optimal combination of minimum irrigation and nitrogen application thresholds. Nine different irrigation and nitrogen application scenarios were evaluated through field comparative experiments. Field measurement data were utilized to simulate root water movement using the HYDRUS model, assessing the effects of various water–nitrogen coupling conditions on water dynamics, nitrogen migration, and root distribution in maize. Additionally, correlation analysis was employed to determine the influence of soil environmental factors on maize yield. The findings of this study offer essential technical insights and theoretical foundations for further understanding the mechanisms by which water–nitrogen interactions impact plant growth, ensuring stable crop yields while minimizing nutrient loss.

## 2. Results

### 2.1. Root Spatial Distribution

**Figure 1:**
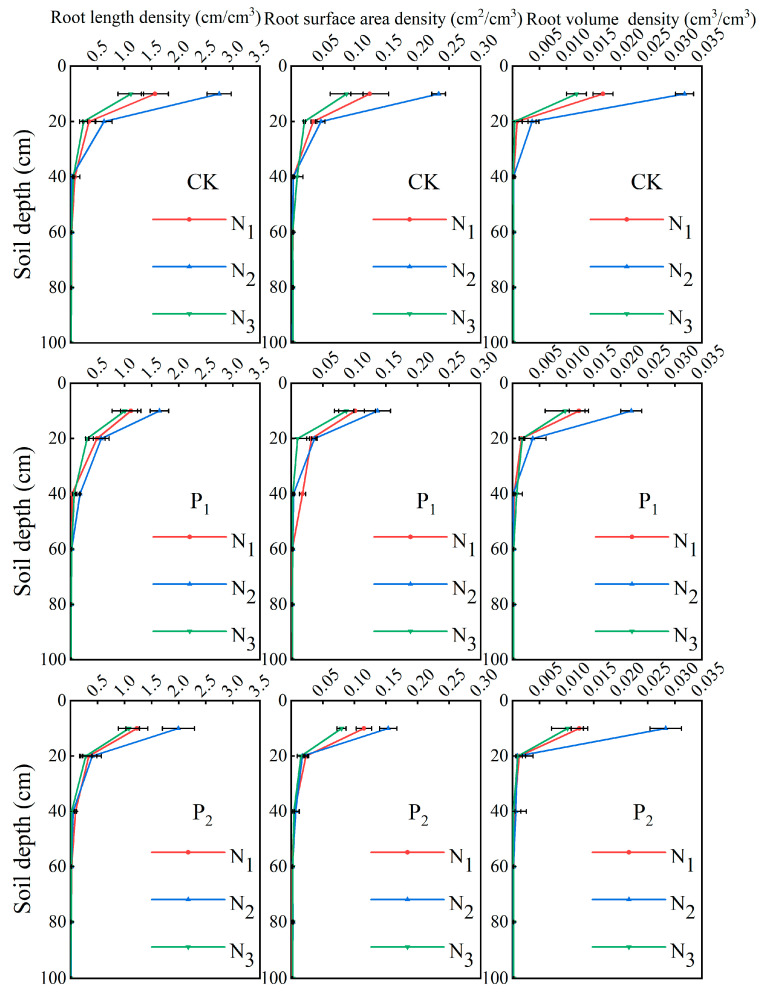
Changes in RLD, RSD, and RVD in the vertical soil profile across treatments at the tasseling stage.

Figure 1 illustrates the vertical distribution of root length density (RLD), root surface density (RSD), and root volume density (RVD) across different soil depths. Root characteristics among various treatments exhibited similar distribution patterns. Following re-watering after drought stress, root systems were primarily concentrated within the 0–30 cm soil layer, accounting for 82.5–87.9% of the total root mass. The highest root morphology parameter values were observed in the 10–30 cm soil layer, with significant variations. At the same nitrogen application level, drought stress at the jointing stage (P_1_) had a more pronounced effect on RLD, RSD, and RVD in the 0–20 cm soil layer compared to drought stress at the tasseling-silking stage (P_2_). Compared to the control group (CK), the maximum values of RLD, RSD, and RVD in P_1_ decreased by 30.6%, 27.0%, and 27.1%, respectively, while reductions of 20.7%, 21.7%, and 15.4% were observed in P_2_. Increasing nitrogen application under the same irrigation conditions altered root morphology, following the trend N_2_ > N_1_ > N_3_. Compared to normal nitrogen application (N_2_), the maximum values of RLD, RSD, and RVD decreased by 38.9%, 35.0%, and 49.6% under low nitrogen (N_1_), while reductions of 49.9%, 51.4%, and 61.4% were recorded under high nitrogen (N_3_). These findings indicate that both high and insufficient nitrogen application inhibited root growth and development.

**Figure 2:**
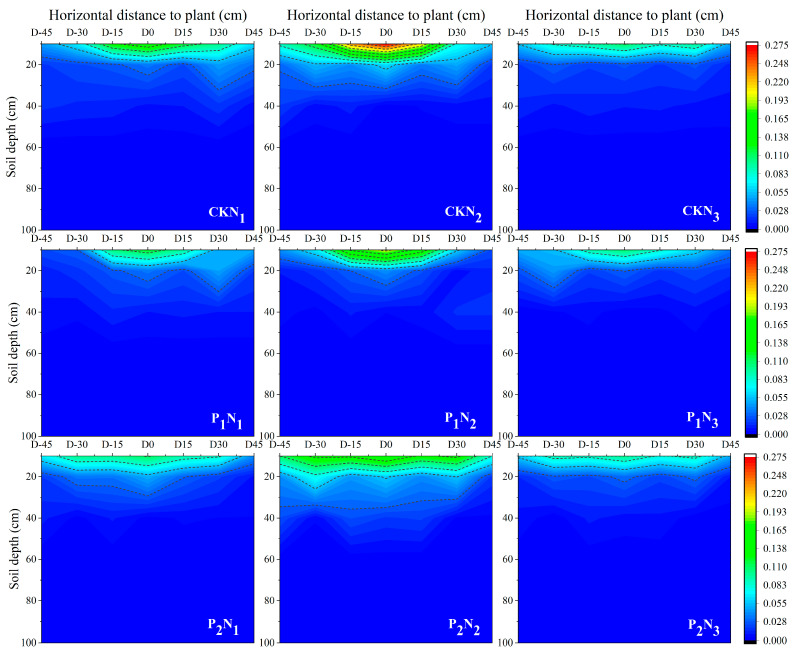
Spatial distribution of root projection area across treatments at the tasseling stage.

In the horizontal direction, root distribution exhibited significant variations depending on water and nitrogen levels as well as horizontal positioning. As shown in Figure 2, under different water treatments, roots in the CK treatment were primarily concentrated within the 0–20 cm soil layer, significantly reducing the projection area as soil depth increased. After drought stress, root distribution in P_1_ and P_2_ treatment were lower than that in CK but still followed a decreasing trend, with roots being more evenly dispersed. Regarding nitrogen treatments, root length density in the surface soil initially decreased and then increased. However, root density under the N_3_ treatment remained lower than that under the N_2_ treatment, with a tendency for roots to extend horizontally. In stress environments, roots exhibited a certain degree of recovery and showed a propensity for horizontal development.

### 2.2. Nitrogen Spatial Distribution

**Figure 3:**
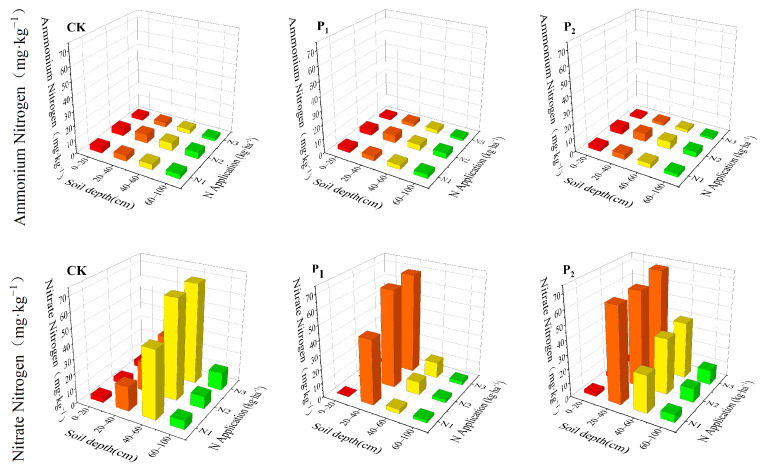
Spatial distribution of nitrate nitrogen and ammonium nitrogen in soil profiles of each treatment at the tasseling stage.

Figure 3 illustrates the vertical distribution of NH_4_^+^-N and NO_3_^−^-N in the 0–100 cm soil profile across different treatments at the tasseling stage of maize. In all treatments, NH_4_^+^-N values were low and exhibited minimal variation. All ammonium values were below 8 mg/kg, and the mean value was about 3 mg/kg. The ammonium nitrogen content followed the trend of N_2_ > N_1_ > N_3_ under different nitrogen treatments, with no significant variations in the vertical soil profile. In contrast, nitrate nitrogen (NO_3_^−^-N) exhibited pronounced dynamic patterns. After drought stress at the jointing stage, peak nitrate nitrogen (NO_3_^−^-N) was concentrated in the 20–40 cm soil layer, whereas for the tasseling-silking stage after drought stress, although the peak value was still 20–40 cm, the nitrate nitrogen content in the 40–60 cm soil layer increased 79% compared to jointing stages, showing a tendency to shift to the deeper soil layer.

### 2.3. Root Water Uptake

**Figure 4:**
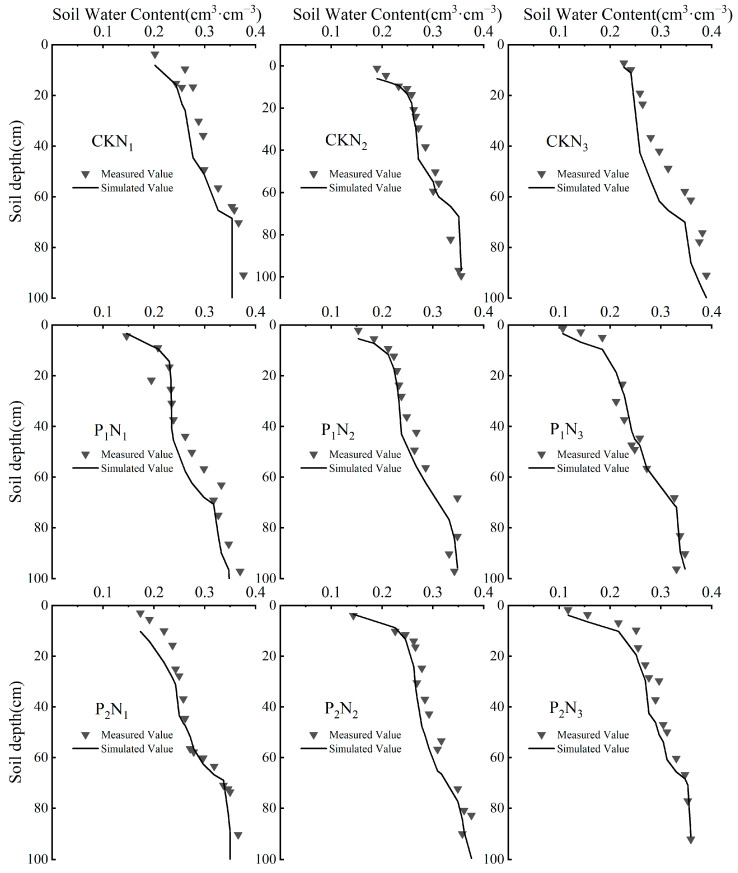
Comparison of actual moisture measured values and simulated values at jointing and grouting.

Soil moisture data obtained during the jointing and tasseling-silking stages were utilized to simulate the maize one-dimensional vertical root water uptake model. The comparison between simulated and measured values is shown in Figure 4. Under different treatments, soil moisture content in the 20 cm layer displayed large variations due to direct irrigation effects. In contrast, moisture fluctuations progressively diminished with soil depth. The 0–40 cm soil layer represented the primary zone for root water absorption, with the average root water uptake in this layer accounting for 69.8%, 79.8%, and 76.5% of the total root water uptake for the N_1_, N_2_, and N_3_ treatments, respectively. The error analysis of the actual measured and simulated soil moisture values for each treatment is shown in Table 1. The RMSE and R^2^ values for the simulated versus measured values were 0.0195 and 0.8832 cm^3^·cm^−3^, respectively. Overall, the model exhibits a satisfactory level of reliability.

### 2.4. Yield and Its Component Factors

Table 2 demonstrates that irrigation levels, nitrogen application rates, and their interaction significantly affect maize yield and its component factors (*p* < 0.01). The influence of irrigation on yield components is more pronounced than that of nitrogen application. The results showed that under the same irrigation conditions, the average yields of CK, P_1_, and P_2_ were 10291, 7691, and 9435 kg/ha, respectively, and the ear weight, 1000-grain weight, and total yield followed the trend N_2_ > N_1_ > N_3_. With increasing nitrogen application, the number of grains per ear initially rises and then declines. At the same nitrogen level, variations in ear length, 1000-grain weight, and yield follow the pattern CK > P_2_ > P_1_, with drought stress at the jointing stage (P_1_) exerting the most significant negative impact on overall yield. Additionally, once nitrogen application reaches a certain threshold, further increases do not lead to substantial yield improvements. The highest yield was recorded in the CKN_2_ treatment at 13,336 kg·ha^−1^, while the lowest yield was observed in the P_1_N_3_ treatment at 4892 kg·ha^−1^.

### 2.5. Correlation Analysis of Crop Roots, Soil Moisture, Nitrogen Content, and Yield

**Figure 5:**
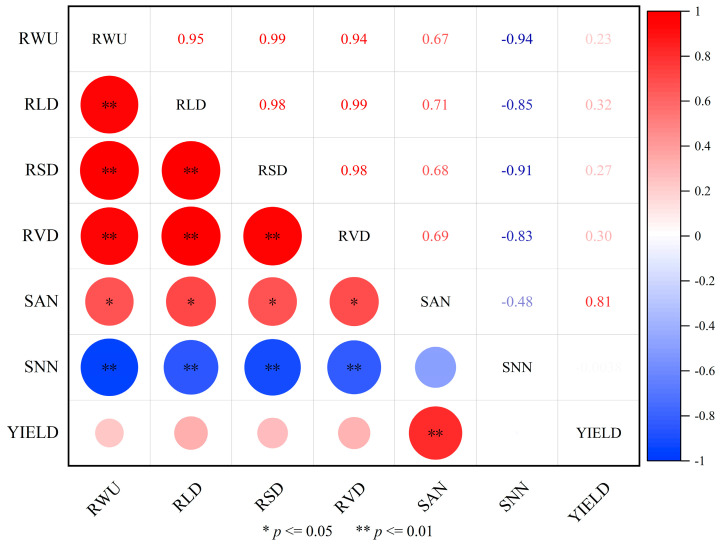
Correlation analysis of root characteristics, nitrogen content, root water uptake, and yield. Note: RLD is root length density, RSD is root surface density, RVD is root volume density, RWU is root water uptake, SAN is soil ammonium nitrogen, SNN is soil nitrate nitrogen.

To investigate the effects of post-drought rewatering during the jointing and tasseling-silking stages on maize root systems and nutrient distribution across different soil layers, a correlation analysis was conducted between root morphology and soil nutrient content. As shown in Figure 5, RLD, RSD, and RVD exhibited significant correlations (*p* < 0.01), demonstrating a strong synergistic relationship among root morphology parameters. A significant negative correlation (*p* < 0.01) was observed between maize root morphology parameters and soil nutrient content. In addition, yield showed a non-significant positive correlation with soil nitrogen levels.

## 3. Discussion

Root system plasticity plays a central regulatory role in maize response to drought stress, with spatial architectural reactions to water and nitrogen inputs influencing subsequent yield formation [31,32,33]. The experimental results indicated that compared with the tasseling-silking stage, corn is more sensitive to drought during the jointing stage, with yield and its components showing significantly greater reductions under jointing-stage drought stress. This differential sensitivity correlates with root spatial architecture: in drought stress treatment, root distribution is mainly concentrated in the 0–40 cm soil layer, accounting for 82.5–87.9% of the total root system, severely inhibiting the development of maize roots. Compared to the CK, the root characteristics of P_1_ showed a more significant decrease compared to P_2_ treatment. Higher irrigation levels significantly enhanced total RLD, RSD, and RVD, as increased soil moisture availability improved root growth and hydraulic conductivity, thereby promoting root development [34], while nitrogen effects exhibited a non-linear response: moderate nitrogen (N_2_) promoted root growth, whereas high nitrogen (N_3_) caused significant declines. Nitrogen application effects revealed unexpected patterns; in different irrigation treatments, the NO_3_^−^-N values in the high nitrogen application treatment N_3_ showed minimal variation; in the 20–40 cm soil layer depth under P_1_ and P_2_ treatments, where the low nitrogen application treatment N1 was 56 mg/kg, N_2_ treatment was 67.4 mg/kg, and N_3_ treatment was 68.9 mg/kg. This suggests that the conventional osmotic stress hypotheses may not fully explain the observed yield suppression in N_3_ treatment [35,36]. Instead, the interaction between irrigation timing and nitrogen mobility appears to be crucial: under CK treatment conditions, the NO_3_^−^-N peak of the high nitrogen application treatment N_3_ shifts to the 40–60 cm soil layer, whereas nitrogen accumulation dominated in the shallow soil layer in the P_1_N_3_ and P_2_N_3_ treatments, which experienced drought stress during the critical fertility period. In addition, NH_4_^+^-N values remained below 8 mg/kg in all treatments, which dispelled concerns about phytotoxic effects. Therefore, we think that the observed yield decline in the high nitrogen application treatment N_3_ does not directly stem from nutrient application more or less, but may stem from a mismatch between irrigation-dependent nitrogen application and the fertility stage of crop demand. These findings underscore the importance of dynamic water–nitrogen management, as crop responses are shaped by real-time soil–plant–atmosphere interactions.

Higher irrigation levels increase the depth of the soil wetting layer, and root distribution exhibits a significant positive correlation with soil moisture content [37]. Studies have shown that 82.5–87.9% of the total root system is concentrated within the 0–40 cm soil layer. Drought stress during the jointing stage (P_1_) has a more pronounced effect on surface roots compared to the tasseling-silking stage (P_2_), as the jointing stage represents a critical phase of crop growth when the root system is highly sensitive to drought stress. In the 0–20 cm soil layer, increased nitrogen application significantly enhanced RLD, RSD, and RVD. Additionally, nitrogen’s influence on root system traits follows a unimodal distribution along the vertical profile, intensifying its effects before declining with increasing soil depth. This nonlinear response is likely associated with the soil moisture gradient, as additional N can only be exploited if there is enough water. The yield and root decline of N_3_ is shown in all treatments. Under conditions of higher moisture content, increased nitrogen application effectively promotes root morphological development, whereas, under drought stress, high nitrogen application may restrict root growth [38,39]. Furthermore, spatial projection of the root system in Figure 5 showed that under different moisture treatments, the projected area showed a trend of vertical extension, and decreased significantly with the increase in soil depth. Under different nitrogen application treatments, the projected areas of both N_1_ and N_3_ treatments were smaller than that of N_2_, showing the trend of N_2_ > N_1_ > N_3_, and the enhanced horizontal extension of roots under the N_3_ treatment may serve as an adaptive strategy for coping with environmental stress. This phenotypic plasticity facilitates the expansion of the rhizosphere, compensating for reduced nutrient absorption efficiency [40,41]. Considering both vertical and horizontal distribution characteristics, roots exhibit enhanced vertical growth under water limitations, while nitrogen imbalance prompts greater horizontal expansion. This “biphasic adaptive strategy” may represent a key survival mechanism for crops subjected to compound stress conditions.

Root system characteristics play a critical role in regulating RWU, and the extent to which RWU meets a crop’s transpiration demand directly influences the assessment of water stress and its severity [42,43]. Based on the spatial distribution of water absorption, the 0–40 cm soil layer serves as the primary water uptake zone for maize roots. The average RWU proportions for the N_1_, N_2_, and N_3_ treatments are 69.8%, 79.8%, and 76.5%, respectively. This pattern is closely associated with the physiological traits of maize’s shallow root system and the nutrient enrichment in the tillage layer. The high variability in soil moisture content within the surface layer (0–20 cm) is primarily attributed to the direct effects of irrigation events. In contrast, the lower variability is observed in deeper soil layers (e.g., 80 cm), which may be due to time-lagged seepage from historical precipitation events being buffered by soil texture. Additionally, the model’s simulation error in the surface soil is slightly higher than in deeper layers, likely due to the heterogeneous composition of surface soil, minor variations in root distribution, and the transient nature of evaporation and infiltration processes.

The optimization of water–nitrogen (W–N) co-management requires alignment between nutrient supply and crop demand dynamics [44,45]. In our research, the experimental data indicated that under identical irrigation conditions, nitrogen application levels consistently followed the hierarchy N_2_ > N_1_ > N_3_ in terms of yield components. Similarly, under the same level of nitrogen application showed a clear gradient CK > P_2_ > P_1_ in yield performance, with drought stress at the jointing stage (P_1_) causing the most severe yield reduction. The absence of crossover effects in the treatment gradient trends suggests that water and nitrogen mainly have independent effects on yield formation and that optimizing irrigation and nitrogen application independently is the main pathway to achieving high yields. Compared to the effects of irrigation levels on yield changes, the impact of nitrogen application rates on yield changes is greater. The average yield difference between N_2_ and N_3_ is 38.9%, while the average difference between irrigation levels is 16.8%, which is consistent with the root morphology analysis. Correlation data showed that RLD, RSD, and RVD were strongly synergistic (*p* < 0.01) but significantly negatively correlated with soil N content. This indicates that the high-nitrogen application treatment (N_3_) may induce an imbalance in root osmotic potential, thereby inhibiting root expansion, despite adequate soil moisture. Unlike the significant W–N interactions reported in previous maize studies [46,47], our results suggest an additive rather than multiplicative effect of water and nitrogen. The 200 kg N/ha threshold in N_2_ treatment likely saturated maize nitrogen demand under non-stress irrigation (CK), rendering additional nitrogen inputs (N_3_) counterproductive due to carbon–nitrogen metabolic trade-offs. Crops experiencing drought stress at the nodulation stage (P_1_) severely affected root development with a 30.6% reduction in RLD compared to CK, which also limited root N uptake. Roots in the high N application treatment (N_3_) under limited irrigation (P_1_/P_2_) were concentrated in the 0–30 cm soil layer, accounting for 82.5–87.9% of the total root system, which reduced the uptake of deeper soil water to some extent and intensified water competition. It may be this decoupling of time phases and spatial mismatch leads to this non-interaction. The contents of soil NH_4_^+^-N and NO_3_^−^-N in this study appear relatively low compared to those in the traditional high-nitrogen planting mode. However, other scholars have obtained similar experimental results. Carciochi observed similar levels of NH_4_^+^-N and NO_3_^−^-N in corn fields under water-saving measures [48]. However, by enhancing root foraging and temporal nitrogen supply to match critical growth stages, yields were improved. Liu also reported a 12–15% increase in maize yield under optimized W–N coupling conditions, even though NO_3_^−^-N levels in the soil remain below 20 mg/kg after harvest [49]. Therefore, our research results highlight the focus of tailored management: in arid and semi-arid areas, ensuring sufficient irrigation at critical stages is a prerequisite for nitrogen optimization, and under conditions of sufficient water sources, nitrogen use becomes the main factor determining yield.

## 4. Materials and Methods

### 4.1. Overview of the Study Area

**Figure 6:**
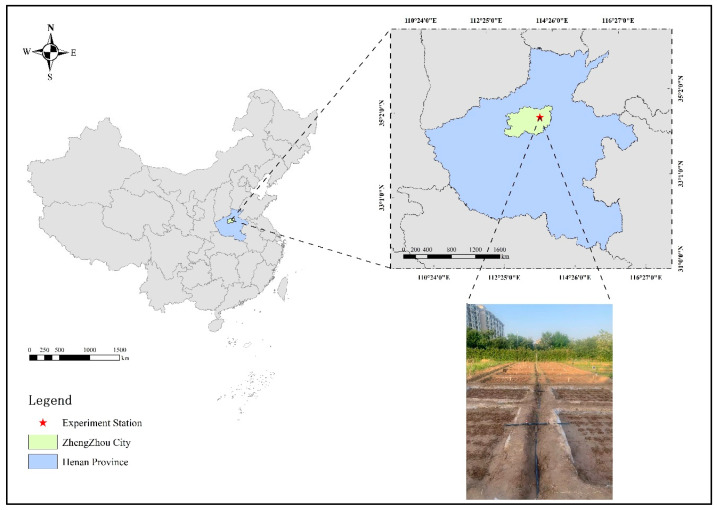
Schematic diagram of the experimental location.

**Figure 7:**
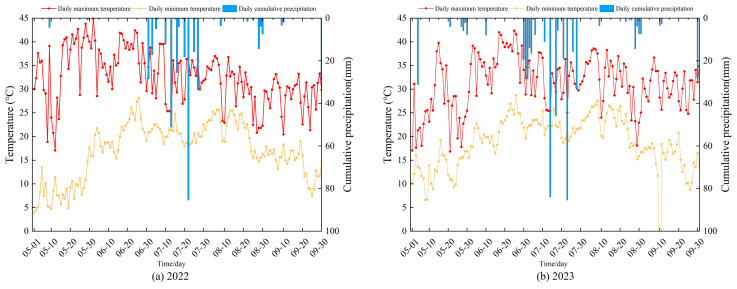
Soil temperature and rainfall during the whole maize growth stages.

The experiment was conducted from June 2022 to September 2023 at the Water-Efficient Agriculture Laboratory (113°47′21″ E, 34°47′21″ N) of North China University of Water Resources and Electric Power in Zhengzhou, China. The geographical layout of the study area is illustrated in Figure 6. The experimental site experiences a temperate continental monsoon climate, characterized by an annual mean temperature of 14.3 °C, 2400 h of sunshine, 637.1 mm precipitation, groundwater depth in the experimental area exceeds 15 m. Approximately 60–70% of annual precipitation occurs between July and September, coinciding with the peak growth stage of maize (*Zea mays* L.). Figure 7 demonstrates the data of rainfall and temperature recorded during the experimental period.

### 4.2. Experimental Materials

The maize variety “*Denghai 605*” was selected for this experiment, which was conducted using a furrow pit trial. The sowing and maturity times varied across the two-year trial cycle: sown on 10 June 2022, with harvest on 22 September, and sown on 6 June 2023, with harvest on 14 September. Wheat was planted during the intervals between corn cycles to maintain continuous land use. Each test plot measured 3 m × 3 m, with plastic isolation membranes installed around the furrow pits to prevent nutrient and water infiltration. A 0.5 m isolation trench was established between adjacent furrow pits. Deep plowing was implemented using a moldboard plough (Model 1LF-440, YTO Corp., Luoyang, China) for inverse tillage at 30 ± 2 cm depth 15 days prior to sowing, disrupting the compacted soil layer formed by long-term shallow tillage to ensure proper root extension and development, followed by surface leveling with a rotary tiller (Model 1GQN-250, 280 ± 5 rpm, YTO Corp., Luoyang, China). Maize seeds were manually sown at a row spacing of 25 cm, plant spacing of 20 cm, and sowing depth of 3–5 cm, resulting in 154 plants per test plot. In each furrow pit (9 m^2^), potassium fertilizer was applied as potassium sulfate (K_2_O 50%) at 400 g per pit (equivalent to 450 kg·ha^−1^), and phosphorus fertilizer as superphosphate (P_2_O_5_ 16%) at 900 g per pit (equivalent to 1000 kg·ha^−1^). Urea (CH_4_N_2_O, 46%) was applied at three nitrogen levels. The total nitrogen fertilizer was split into two equal doses: 50% as a basal application and 50% as a topdressing. For the basal dose, urea was broadcast evenly into the furrows during sowing and incorporated into the top 10 cm soil layer by manual raking. The remaining 50% of urea was surface-applied at the jointing stage (BBCH 30) and immediately irrigated with 20 mm water to facilitate infiltration into the root zone. Phosphorus fertilizer and potassium fertilizer were applied in full as basal doses, thoroughly mixed with the soil prior to sowing.

The soil type in the experimental field was loam, with clay (<0.002 mm), silt (0.002–0.02 mm), and sand (0.02–2 mm) comprising 3.8%, 43.1%, and 53.1%, respectively. The soil exhibited a bulk density ranging from 1.46 to 1.55 g/cm^3^ and an electrical conductivity of 0.32 μS·cm^−1^. The basic physicochemical properties of the soil layers from 0 to 100 cm are detailed in Table 3.

### 4.3. Experimental Design

Different levels of irrigation and nitrogen fertilization have been applied in the experiment. The irrigation has been tested at three levels: control (CK), drought stress during jointing stage (P_1_), and drought stress at the tasseling-silking stage (P_2_). In the control treatment and the non-stress periods, soil moisture was kept at 75–100% field capacity. For treatments P_1_ and P_2_, drought stress was applied during their respective growth phases by maintaining soil moisture at 50–60% of field capacity. This was controlled by means of soil moisture sensors (Model ZL-06, INSENTEK Corp., Hangzhou, China), with real-time data collection over 24 h. Additionally, weekly measurements were performed using the soil drying method to correct discrepancies. In addition, three nitrogen levels have been applied: N_1_ (low nitrogen, 100 kg·N ha^−1^), N_2_ (normal nitrogen, 200 kg N·ha^−1^), and N_3_ (high nitrogen, 300 kg·N ha^−1^). The experimental design is presented in Table 4.

Before sowing, soil water content in all plots was adjusted to 70–75% of field capacity to ensure optimal seedling establishment and prevent excessive wetness or dryness, which could interfere with germination. Following sowing, soil moisture levels were monitored continuously using soil moisture sensors (Model ZL-06, INSENTEK Corp., Hangzhou, China), with real-time data collection over 24 h. Additionally, weekly measurements were performed using the soil drying method to correct discrepancies. This ensured that soil moisture across all treatments remained within 70–75% of field capacity before entering the pre-treatment growth stages. The maize growth stages were classified using the BBCH code. In this experiment, P_1_ (BBCH 30–39) featured stem elongation with 2–3 visible internodes, accompanied by accelerated plant growth and leaf expansion. P_2_ (BBCH 51–59) was marked by full kernel development, initial starch accumulation in the endosperm exhibiting a milky consistency, and the formation of translucent, moisture-rich grain structures. A growth stage was considered reached when more than 50% of the plants in a plot exhibited the corresponding characteristic traits.

### 4.4. Sampling, Measurement, and Calculation Methods

#### 4.4.1. Root Sample Collection

During the reproductive period of maize, soil cores were collected weekly using a screw auger (6.8 cm inner diameter, 7 cm outer diameter). Soil cores were taken down to 100 cm deep at 10 cm intervals close to each sampled plant for root parameter analysis. Each soil sample was transferred to a 0.5 mm sieve and the soil was washed off under low pressure, leaving only the root segments. The root length density (RLD), root surface area density (RSD), and root volume density (RVD) were analyzed using the root image analysis system (Win RHIZO Pro 2007d, Regent Instruments Inc., Quebec, QC, Canada) based on the images using the roots scanner (Epson Expression 1600 pro, Model EU-35,Yamagata, Japan). Root eigenvalue density was calculated as the ratio of all root segments in each soil core to the volume of the core. Root densities of samples taken from each soil profile were normalized by the total root system from all samples taken from the soil profile.

#### 4.4.2. Soil Nutrient Determination and Analysis

Soil samples were collected weekly using a soil auger during the jointing stage (BBCH 30–39) and tasseling-silking stage (BBCH 51–59) of maize. Sampled soil profiles were taken at depths of 0–100 cm. Soil samples were air-dried and passed through a 0.149 mm sieve, then leached in KCL solution (2 mol/L KCL solution, 5 g of soil sample, soil-liquid ratio 1:10), shaken for 2 h and filtered, and the resulting leachate was analyzed for nitrate nitrogen and ammonium nitrogen contents using the AA3 continuous flow analyzer (SEAL Analytical GmbH Corp., Norderstedt, Germany).

#### 4.4.3. Yield and Components

During the maize harvest, ten plants were randomly chosen from each experimental plot for yield analysis. Parameters such as ear weight, number of grains, and hundred-grain weight were measured after threshed and dried. Heat-treated at 105 °C for 30 min to deactivate enzymes in a vacuum oven, followed by drying at 75 °C for 3–4 h until constant weight. The yield per unit area was subsequently calculated.

### 4.5. Model Construction

This study used the HYDRUS-1D numerical model to simulate soil moisture dynamics and root water uptake under experimental conditions. The simulation model consisted of three sub-models: soil moisture dynamics model, root water uptake model, and evapotranspiration and transpiration model. These three sub-models are coupled with each other and work together.

#### 4.5.1. Soil Moisture Dynamics Model

Soil moisture kinematic equations were determined using the Richards equation with source-sink terms.

(1) $$\frac{\partial \theta }{\partial t}=\frac{\partial }{\partial z}\left[D\left(\theta \right)\frac{\partial \theta }{\partial z}\right]-\frac{\partial K\left(\theta \right)}{\partial z}-S\left(z,t\right)$$

where z is vertical coordinate (cm), t  is time (h), θ is volumetric soil moisture content (%), $D\left(\theta \right)$ indicates the soil water diffusivity (cm^3^·h^−1^), $K\left(\theta \right)$ represents unsaturated hydraulic conductivity, S is the root water uptake term (h^−1^), defined as the rate of water absorption by roots from a unit volume of soil per unit time.

Soil moisture characteristic curves were fitted using the Van−Genuchten model.

(2) $$\theta \left(h\right)=\theta_{r}+\frac{\theta_{s}-\theta_{r}}{{\left(1+{\left|\alpha h\right|}^{n}\right)}^{m}},\;(h\leq 0)$$

(3) $$K\left(h\right)=K_{s}(\frac{\theta -\theta_{r}}{\theta_{s}-\theta_{r}}{)}^{l}\{1-{\left[1-{\left(\frac{\theta -\theta_{r}}{\theta_{s}-\theta_{r}}\right)}^{\frac{1}{m}}\right]}^{n}{\}}^{2}$$

where h is pressure head (cm), h = 0 indicates saturated soil conditions. $\theta_{r}$ is residual water content (cm^3^/cm^−3^), while $\theta_{s}$ corresponds to saturated water content (cm^3^/cm^−3^). The parameters α, n, and m characterize the soil model, with m defined as 1 − 1/n. $K_{s}$ is saturated hydraulic conductivity (cm/d), l is empirical fitting parameter, typically assigned a mean value of 0.5.

#### 4.5.2. Root Water Uptake Model

In the soil moisture kinematic equations, the source-sink term $S\left(z,t\right)$ accounts for root water uptake. This process is typically characterized using the Feddes model expressed as follows:

(4) $$S\left(z,t\right)=\alpha \left(h\right)b\left(z\right)T_{P}$$

where $S\left(z,t\right)$ is potential root water uptake rate (h^−1^), $\alpha \left(h\right)$ represents the water stress response function quantifying soil water potential effects on uptake, $b\left(z\right)$ the root length density distribution function, $T_{P}$ is potential transpiration rate (cm·h^−1^). The functional form of $\alpha \left(h\right)$ is defined as follows:

(5) $$\alpha \left(h\right)=\left\{\begin{array}{c} 0,\;\;\;\;\;\;\;\;\;\;\;\;\;\;h>h_{1}\;or\;h\leq h_{4}\;\\ \frac{h-h_{1}}{h_{2}-h_{1}},\;\;\;\;\;\;\;\;\;\;\;\;h_{2}<h\leq h_{1}\\ 1,\;\;\;\;\;\;\;\;\;\;\;\;\;\;h_{3}<h\leq h_{2}\\ \frac{h-h_{4}}{h_{3}-h_{4}},\;\;\;\;\;\;\;\;\;\;\;\;\;\;h_{4}<h\leq h_{3} \end{array}\right.$$

where $h_{1}$ is the pressure head at anaerobic stress onset (cm), $h_{2}$ and $h_{3}$ are the optimal growth pressure head range (cm), $h_{4}$ is the wilting point pressure head (cm).

#### 4.5.3. Evapotranspiration Model

Daily precipitation, mean air temperature, relative humidity, sunshine duration, and wind speed were recorded using an on-site weather station (RX3000, HOBO, Annapolis, MD, USA). Evapotranspiration (*ETo*, mmd^−1^) was calculated using the Penman–Monteith equation:

(6) $${ET}_{O}=\frac{0.408∆(R_{n}-G)\gamma \frac{900}{T+273}u_{2}(e_{S}-e_{a})}{∆+\gamma (1+0.34u_{2})}$$

where $R_{n}$ is crop surface net radiation (MJ m^−2^d^−1^), G is soil heat flux (MJ m^−2^d^−1^), T is the mean air temperature (°C), $u_{2}$ is wind speed at 2 m height (m·S^−1^), $\left(e_{S}-e_{a}\right)$ is vapor pressure deficit (Kpa), ∆ slope of saturation vapor pressure-temperature curve (kPa·°C^−1^), and γ represents the psychrometric constant (kPa·°C^−1^).

Crop potential evapotranspiration (${ET}_{c}$) was determined using the single crop coefficient approach. The Beer’s law was applied to partition ${ET}_{c}$ into potential transpiration ($T_{P}$) and soil evaporation ($E_{P}$).

(7) $${ET}_{c}=k_{c}{ET}_{O}$$

(8) $${ET}_{c}=T_{P}+E_{P}={ET}_{c}\left(1-e^{-0.6LAI}\right)+{ET}_{c}e^{-0.6LAI}$$

where $k_{c}$ is actual crop coefficient determined following Reference [50], LAI is the leaf area index.

#### 4.5.4. Model Parameters

The model parameters were obtained from measurement data collected during the 2022–2023 experiment, including soil moisture, soil particle composition, and bulk density. Other soil hydraulic parameters were initially referenced from the model’s soil hydraulic parameter database. These parameters were subsequently calibrated using the inverse method, and the final calibrated values are presented in Table 5.

#### 4.5.5. Boundary Conditions

Initial soil moisture and nitrogen content are assumed to be uniformly distributed over the study area. The initial conditions are expressed as follows:

(9) $$\theta \left(r,z,t\right)=\theta_{k}\;\;\;\;\;\;\;\;\left(0\leq r\leq L,\;\;0\leq Z\leq H,\;\;t=0\right)$$

where $\theta_{k}$ is the initial volumetric water content of the soil (cm^3^/cm^−3^), r is the radial coordinate (cm), z is the vertical coordinate, downward positive (cm), L is the horizontal distance of the simulated area, 0≤L≤300 cm, H is the depth of the soil, simulating the depth of the calculation area, 0≤H≤100 cm.

For the modeled area, the upper boundary is the atmospheric boundary, and the side boundaries (left and right boundaries) are designated as zero flux boundaries, indicating that no material moves across these boundaries. The lower boundary, which considers groundwater burial depths greater than 10 m, was set as a free-drainage boundary, allowing natural movement of water and solutes.

Upper boundary conditions:

(10) $$-K\left(h\right)\frac{\partial h}{\partial z}-K\left(\theta \right)=\sigma \left(t\right)\;\;\;\;\;\;\;\;\;\;\;\;\;\;\;\;\;\left(0\leq r\leq 300,\;\;z=0,\;\;0<t<T\right)$$

**Figure 8:**
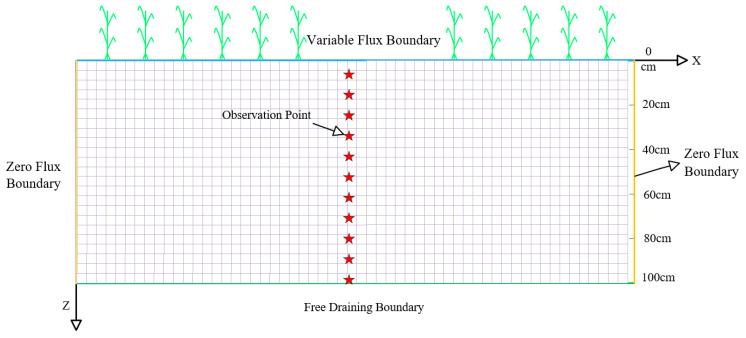
The geometric structure and boundary conditions of the model.

Lower boundary conditions:

(11) $$\frac{\partial h}{\partial z}=0,\;\;z=100,\;\;t\geq 0$$

where $\sigma \left(t\right)$ is the constant flow boundary flux during irrigation (3 cm/d). The model geometry and boundary conditions are shown in Figure 8.

### 4.6. Data Analysis

All experimental data were processed and analyzed using Excel 2019. SPSS 25 (SPSS Inc., Chicago, IL, USA) was used for statistical analysis. The LSD Test and Duncan’s Multiple New Range Test were conducted to evaluate significant differences between samples. The Kolmogorov–Smirnov test was applied to assess data normality. If the data followed a normal distribution, ANOVA was used with a significance level of 5%. For non-normally distributed data, non-parametric tests were employed. All figures were generated using Origin Pro 2022 (OriginLab Corp., Redmond, MA, USA).

## 5. Conclusions

1. The jointing stage represents a critical growth stage for maize root systems. Drought stress during this stage causes more severe impacts on root architecture compared to the tasseling-silking stage. Implementing combined precision irrigation and dynamic nitrogen supplementation strategies during jointing can optimize root configuration while balancing yield improvement with environmental sustainability.
2. Maize roots employ a biphasic adaptive strategy, dynamically balancing vertical deepening and horizontal expansion under environmental stress, reinforcing vertical growth under water limitations while promoting horizontal expansion under nitrogen imbalance. This coordinated vertical–horizontal response mechanism demonstrates the plant’s adaptive capacity to challenging environmental conditions.
3. The HYDRUS-based one-dimensional vertical root water uptake model effectively simulated soil water, with all R^2^ values exceeding 0.8. This confirms its reliability in modeling soil water movement patterns.

## Figures and Tables

**Table 1:** Error analysis of measured and simulated soil moisture values of treatments at tasseling and staminate spatula stages.

Treatment	MAE	RMSE	R^2^
CKN_1_	0.0164	0.0191	0.874
CKN_2_	0.0109	0.0289	0.915
CKN_3_	0.0342	0.0153	0.892
P_1_N_1_	0.0119	0.0169	0.874
P_1_N_2_	0.0209	0.0177	0.892
P_1_N_3_	0.0087	0.0262	0.895
P_2_N_1_	0.0134	0.0154	0.903
P_2_N_2_	0.0121	0.0175	0.840
P_2_N_3_	0.0374	0.0189	0.863

**Table 2:** Effects of water and nitrogen coupling on yield and yield components of maize in 2023.

Treatment	Ear Weight (g)	Number of Grains	Thousand-Grain Weight (g)	Yield (kg·ha^−1^)
CKN_1_	96.9 b	394.0 c	310.5 b	9785 b
CKN_2_	129.2 a	524.2 a	316.8 a	13,336 a
CKN_3_	78.9 c	334.1 cd	293.1 c	7751 c
P_1_N_1_	66.5 cd	287.0 d	292.2 c	6692 d
P_1_N_2_	111.5 a	436.3 b	329.6 a	11,489 b
P_1_N_3_	48.5 d	298.0 d	205.6 e	4892 e
P_2_N_1_	90.9 b	325.6 cd	341.1 a	9049 c
P_2_N_2_	122.5 a	454.4 b	339.2 a	12,216 a
P_2_N_3_	75.2 c	420.0 b	211.7 e	7042 d

Note: Different letters within a date indicate significant differences (*p* < 0.05) between treatments.

**Table 3:** Soil physical and chemical properties.

Soil Layer (cm)	Soil Bulk Density (g·cm^−3^)	Soil Organic Matter (g·kg^−1^)	Field Water Retention (V/V%)	Total N (g·kg^−1^)	Ammonium Nitrogen (mg·kg^−1^)	Nitrate Nitrogen (mg·kg^−1^)	Total P (g·kg^−1^)	pH
0–20	1.46	16.1	34.2	0.89	7.43	8.15	0.75	6.7
20–40	1.48	14.5	35.1	0.93	6.71	7.75	0.94	
40–60	1.51	5.6	34.7	0.67	5.72	7.03	0.56	
60–80	1.55	3.4	34.5	0.45	5.55	6.51	0.38	
80–100	1.55	2.1	34.6	0.22	4.21	6.14	0.19	

**Table 4:** Experimental design of water stress and salt stress.

Treatment	Soil Moisture		N Application (kg·ha^−1^)
	P_1_	P_2_	
CKN_1_	75–100%	75–100%	100
CKN_2_			200
CKN_3_			300
P_1_N_1_	50–60%	75–100%	100
P_1_N_2_			200
P_1_N_3_			300
P_2_N_1_	75–100%	50–60%	100
P_2_N_2_			200
P_2_N_3_			300

Note: CK (control check, full irrigation), P_1_ (drought at jointing stage), P_2_ (drought at tasseling-silking stage); N_1_/N_2_/N_3_ (nitrogen levels).

**Table 5:** Parameters of soil hydraulic properties.

Soil Layer (cm)	$\theta_{r}$ (cm^3^·cm^−3^)	$\theta_{s}$ (cm^3^·cm^−3^)	α (1·cm^−1^)	n	K (cm·d^−1^)
0–20	0.0371	0.3105	0.0128	1.3941	35.60
20–40	0.0386	0.3209	0.0136	1.1392	25.13
40–60	0.0394	0.3752	0.0181	1.0633	35.56
60–80	0.0342	0.3578	0.0134	1.3855	34.19
80–100	0.0360	0.3809	0.0152	1.5630	45.56

## Data Availability

Data are contained within the article.
